# Functional significance of phylogeographic structure in a toxic benthic marine microbial eukaryote over a latitudinal gradient along the East Australian Current

**DOI:** 10.1002/ece3.6358

**Published:** 2020-05-21

**Authors:** Arjun Verma, David J. Hughes, D. Tim Harwood, David J. Suggett, Peter J. Ralph, Shauna A. Murray

**Affiliations:** ^1^ Climate Change Cluster University of Technology Sydney Ultimo NSW Australia; ^2^ Cawthron Institute Nelson New Zealand

**Keywords:** functional traits, harmful algal blooms, phylogeography, population ecology, protists

## Abstract

Genetic diversity in marine microbial eukaryotic populations (protists) drives their ecological success by enabling diverse phenotypes to respond rapidly to changing environmental conditions. Despite enormous population sizes and lack of barriers to gene flow, genetic differentiation that is associated with geographic distance, currents, and environmental gradients has been reported from planktonic protists. However, for benthic protists, which have reduced dispersal opportunities, phylogeography and its phenotypic significance are little known. In recent years, the East Australian Current (EAC) has intensified its southward flow, associated with the tropicalization of temperate waters. Benthic harmful algal species have been increasingly found in south‐eastern Australia. Yet little is known about the potential of these species to adapt or extend their range in relation to changing conditions. Here, we examine genetic diversity and functional niche divergence in a toxic benthic dinoflagellate, *Ostreopsis* cf. *siamensis,* along a 1,500 km north–south gradient in southeastern Australia. Sixty‐eight strains were established from eight sampling sites. The study revealed long‐standing genetic diversity among strains established from the northern‐most sites, along with large phenotypic variation in observed physiological traits such as growth rates, cell volume, production of palytoxin‐like compounds, and photophysiological parameters. Strains from the southern populations were more uniform in both genetic and functional traits, and have possibly colonized their habitats more recently. Our study reports significant genetic and functional trait variability in a benthic harmful algal species, indicative of high adaptability, and a possible climate‐driven range extension. The observed high trait variation may facilitate development of harmful algal blooms under dynamic coastal environmental conditions.

## Graphical abstract

Genetic diversity and phenotypic diversity in marine microbial eukaryotic populations are critical for adaptability, range expansion, and potential bloom formation. In this study, we report significant functional trait variability in a benthic harmful algal species, indicative of high adaptability, and a possible climate‐driven range extension along the East Australian Current region in the South Pacific. The observed high trait variation may facilitate development of harmful algal blooms under dynamic coastal environmental conditions.

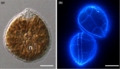

## 1 INTRODUCTION

Microbial eukaryotes (protists) are an essential component of marine ecosystems, driving productivity and biogeochemical cycles (Falkowski et al., 2004). Despite this, our understanding of processes shaping their genotypic and phenotypic diversity remains poor. Protistan populations were originally thought to be largely homogenous, having high dispersal capabilities and fast replication rates, resulting in cosmopolitan distributions with no geographically related population structures (De Wit & Bouvier, 2006; Finlay, 2002). However, recent studies have shown phylogeographic structure to be reflective of historical (dispersal limitation), ecological (divergence resulting from local selection), and/or local adaptive conditions (trait‐based niches) (Casteleyn et al., 2010; Ellison et al., 2011; Litchman & Klausmeier, 2008; Litchman, Klausmeier, Schofield, & Falkowski, 2007; Nagai et al., 2007; Tahvanainen et al., 2012). This suggests that marine microbial eukaryotes should be considered as consortia of genetically structured “meta‐populations” with low dispersal rates (isolation by physical and/or ecological barriers), rather than single panmictic populations (Casabianca et al., 2011; Sjöqvist, Godhe, Jonsson, Sundqvist, & Kremp, 2015).

The ecological success of such populations living in shifting environments is hypothesized to be driven by phenotypic diversity of functional traits (Chevin, Collins, & Lefèvre, 2013). Phenotypic variability among populations could buffer immediate detrimental effects of environmental fluctuations, with more diverse populations expected to buffer changing conditions more efficiently than their uniform counterparts (Bell & Collins, 2008; Kearney, Simpson, Raubenheimer, & Helmuth, 2010; Kremp et al., 2012, 2016). Specifically, harmful algal blooms (HABs) that exhibit substantial intraspecific trait variation are seen as outcomes of population‐scale adaptive strategies requiring the concentrated efforts of numerous cells (Driscoll, Hackett, & Ferrière, 2016; Gilbert, 2016; Rengefors, Kremp, Reusch, & Wood, 2017; Smayda, 1997; Thornton, 2002). Thus, investigating mechanisms of phenotypic variation in such species will expand our understanding of adaptation, and also enable us to predict the dynamics of future populations under changing climatic regimes (Chevin et al., 2013; Collins, Rost, & Rynearson, 2014; Suggett, Warner, & Leggat, 2017).

**FIGURE 1:**
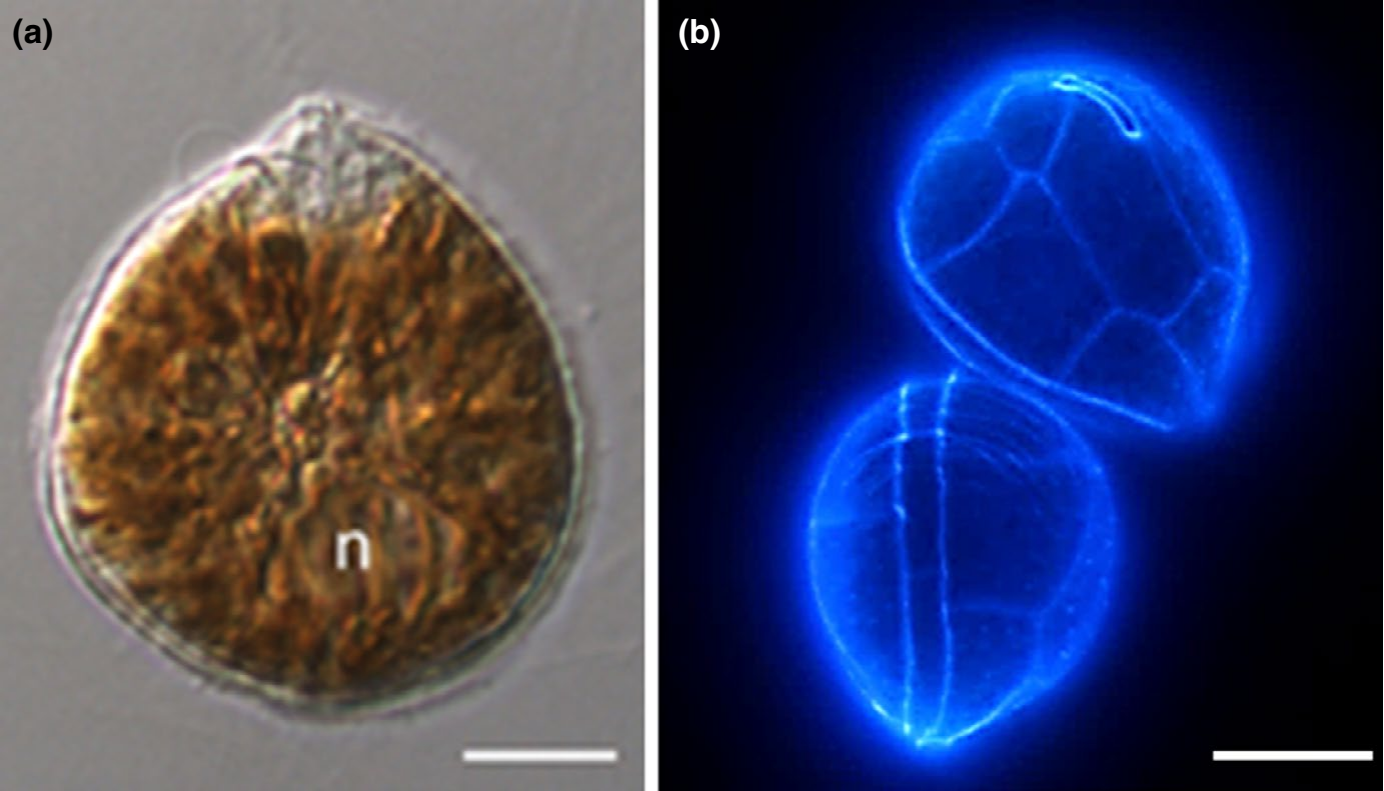
Micrographs of *Ostreopsis* cf. *siamensis* observed in (a) light microscopy (modified from Verma, Hoppenrath, Harwood, et al., 2016); (b) cell wall (theca) visualized by epifluorescence microscopy. Scale bar represents 10 μm. n represents nucleus

In the southeast coastal region of Australia, the East Australian Current (EAC) runs from north to south, redistributing warm tropical waters from the Coral Sea into the temperate Tasman Sea, thereby developing a complex coastal ecosystem along the coast. The EAC has experienced a southern range extension of approximately 350 kilometers in the recent decades, and a significant increase in ocean temperature at the mid‐latitudes of up to 2.0°C, making it a global “hot spot” of climate variation (Ridgway, 2007; Suthers et al., 2011). In recent years, HAB species have been found from the EAC region, with increasing reports of these species being linked to seafood poisoning events and mortalities in fish (Ajani et al., 2016). Among them, *Ostreopsis* cf. *siamensis* (Figure 1) is a cosmopolitan benthic dinoflagellate, found in subtropical and temperate waters (Rhodes, 2011). *O*. cf*. siamensis* is known to cause harmful blooms by producing highly toxic palytoxin‐like compounds (PLTX, C_129_H_223_N_3_O_54_) which cause large‐scale mortalities of benthic organisms, human skin and eye irritations, and illnesses via the consumption of contaminated seafood (Deeds & Schwartz, 2010; Shears & Ross, 2009; Usami et al., 1995). In the EAC region, *O*. cf. *siamensis* has been reported from temperate estuaries and subtropical coral reefs, yet its distribution and functional diversity are not known (Verma, Hoppenrath, Dorantes‐Aranda, Harwood, & Murray, 2016; Verma, Hoppenrath, Harwood, et al., 2016). Thus, the potential of such benthic HAB species to adapt or extend its range in relation to changing conditions is not fully understood.

Here, we assessed the functional and genetic diversity among *O*. cf. *siamensis* strains established from eight sampling sites along a 1,500 km north–south gradient, ranging from subtropical to temperate coastal regions of the EAC. We first determined long‐standing genetic variability and phylogeographic trends among these strains based on ribosomal gene phylogeny and haplotype networks. Then, we examined phenotypic variation in growth rates, cell volume, light harvesting, and utilization parameters and production of PLTX‐like compounds. We observed that populations of this benthic HAB species in the northern subtropical regions are likely to harbor more genetic diversity and phenotypic variation compared to the more “uniform” populations in the southern temperate regions, possibly due to their recent colonization caused by the southward intensification of the EAC. Results from this study provide novel insights into the phylogeographic structure and large phenotypic trait variation within benthic HAB populations along the EAC, which potentially facilitate bloom development under dynamic environmental conditions and promote the success of HABs during global environmental change.

## 2 METHODS

### 2.1 Sample collection and cultures

**FIGURE 2:**
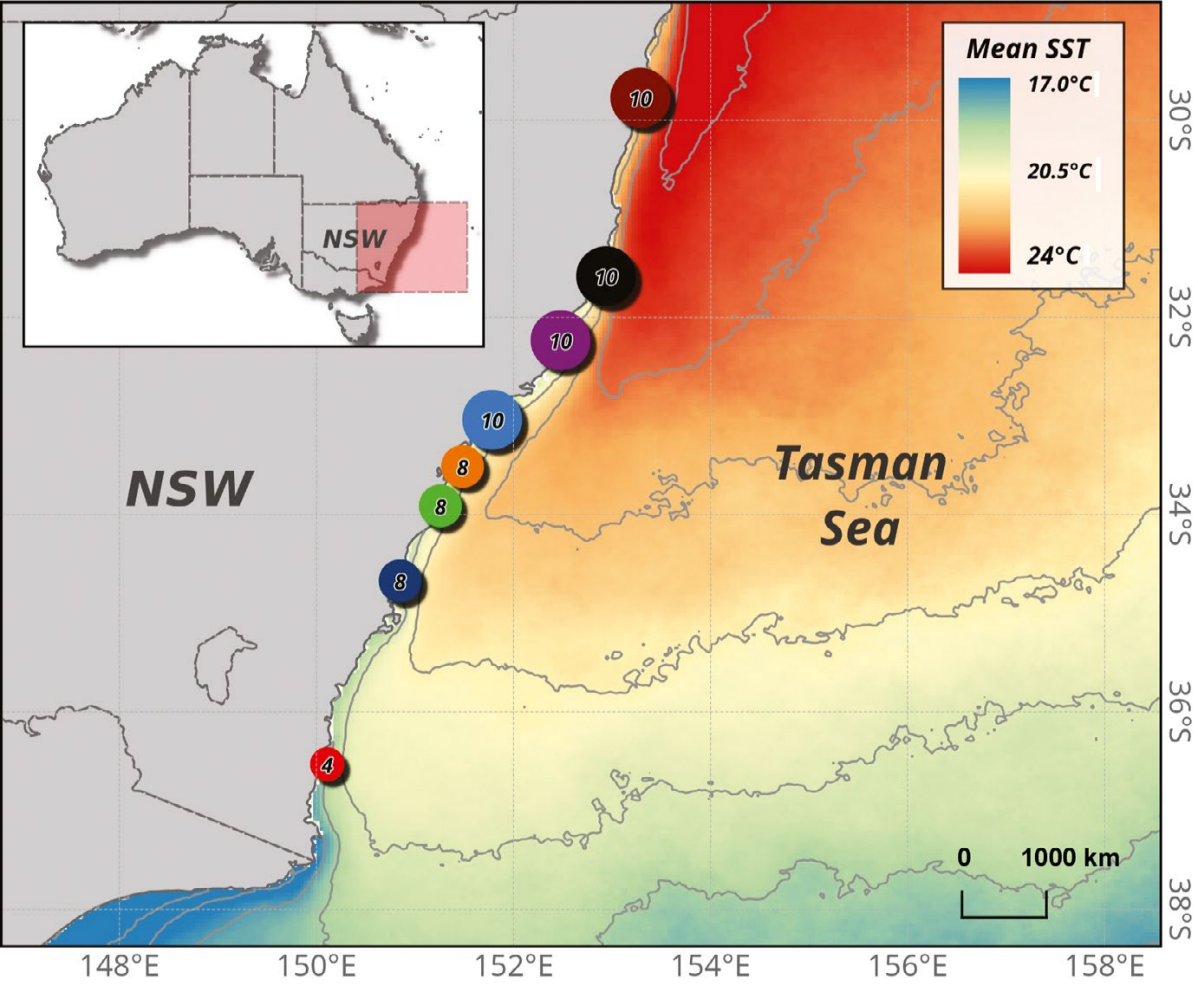
Map of the southeastern coastline of Australia showing sampling locations; Minnie waters (MW), Bonny Hills (BH), Wallis Lake in Forster (FR), Lake Macquarie (LM), Patonga creek (PC), Gordons Bay (GB), Kiama (KM), and Merimbula lake inlet (MER). Numbers inside the circle represent the number of strains that were established from the sampling site. Isothermal lines represent the mean sea surface temperature from 2012 to 2017 varying north to south from 17 to 24°C by a gradient of 1°C

Macroalgal and seagrass samples were collected from eight shallow intertidal sites spanning five bioregions along the New South Wales coast: Tweed‐Moreton, Manning shelf, Hawkesbury shelf, Batemans shelf, and Twofold shelf bioregions (Ajani et al., 2013; Roy et al., 2001), as listed in Table 1 between April and July 2014 (Figure 2). Samples were shaken vigorously to detach the epiphytic microalgal cells, and *Ostreopsis* cells were identified microscopically from the epiphytic suspension. Nonaxenic monoclonal cultures were established according to single cell isolation protocol as described in Verma, Hoppenrath, Harwood, et al. (2016). A total of 68 strains were established and were grown in 5× diluted f/2 medium (Guillard & Ryther, 1962) at 18 ± 1°C and salinity of 35 PSU, under an irradiance of 60 ± 15 µmol photons m^−2^ s^−1^ set to a 12:12‐hr (light: dark) photoperiod, in 70ml sterile tissue culture flasks, up to a volume of 50 ml (Becton Dickinson). Although irradiance measurements showed that the light field was uniform, positions of the flasks were changed randomized every other day. Cultures were maintained in semicontinuous batch mode and diluted to maintain exponential growth as per Hennige, Suggett, Warner, McDougall, and Smith (2009).

**TABLE 1:** Summary of the sampling sites and genetic diversity of *Ostreopsis* cf. *siamensis* strains in this study

Site	Lat; Long	Bioregion	Macrophyte species	Sampling period	Temp (°C)	Salinity (PSU)	Isolates established	Mean pair wise differences	Nucleotide diversity (Average over loci)	No. of polymorphic sites (*S*)	Haplotype diversity (*H*_d_)	No. of haplotypes
Minnie Water (MW)	29.77°S 153.29°E	Tweed‐Moreton shelf	*Hormosira banksii*	July 2014	18	35	10	6.08 (3.17)	0.005 (0.003)	20	0.96 (0.06)	8 [MW1, MW2, MW3, MW4(2),^a^ MW5, MW7, MW8, MW10(2)]
Bonny Hills (BH)	31.58°S 152.82°E	Tweed‐Moreton shelf	*Hormosira banksii*	July 2014	18.5	34	10	3.04 (1.73)	0.002 (0.001)	9	0.53 (0.18)	4 [BH1, BH2, BH3, BH10(7)^a^]
Wallis Lake, Forster (FR)	32.23°S 152.48°E	Manning shelf	*Zostera* sp.	July 2014	19	34	10	2.09 (1.27)	0.0017 (0.0012)	6	0.73 (0.12)	4 [FR1(5),^a^ FR2(2), FR4, FR7(2)]
Lake Macquarie (LM)	33.09°S151.88° E	Hawkesbury shelf	*Ecklonia* sp.	June 2014	17	34	10	0.4 (0.4)	0.00034 (0.00039)	2	0.38 (0.18)	3 [LM2, LM3, LM5(8)^a^]
Patonga Creek (PC)	33.51°S 151.28° E	Hawkesbury shelf	*Sargassum* sp.; *Phyllospora* sp.	May 2014	18	35	8	0.68 (0.57)	0.00057 (0.00055)	2	0.61 (0.16)	3 [PC3, PC7(5),^a^ PC10(2)]
Gordons Bay (GB)	33.91°S 151.26°	Hawkesbury shelf	*Sargassum* sp.	April 2014	18	34	8	1.68 (1.09)	0.0014 (0.0005)	6	0.75 (0.14)	4 [GB2(4),^a^ GB5(2), GB14, GB16]
Kiama (KM)	34.67°S 150.85°E	Batemans shelf	*Phyllospora* sp.; *Sargassum* sp.	July 2014	18	34	8	0.5 (0.47)	0.00042 (0.00046)	2	0.25 (0.18)	2 [KM3, KM8(7)^a^]
Merimbula Lake Inlet (MER)	36.53°S 149.54°E	Twofold shelf	*Zostera* sp.	April 2014	19	28	4	1 (0.83)	0.00085 (0.00084)	2	0.5 (0.265)	2 [MER19(3), MER24(1)^a^]

Note

Standard error estimate(s) are shown in brackets and were obtained by a bootstrap procedure (1,000 replicates). Strain codes are representatives of the various haplotypes. Number in the bracket represents the number of isolates that belong to the representative haplotype.

^a^ The strain codes and the number of strains from each location that represent the common haplotype.

### 2.2 DNA sequencing and molecular analyses

DNA was extracted from 10 ml of cultures using 3% cetyl trimethylammonium bromide (CTAB) extraction buffer (100 mM Tris‐HCl pH 8; 20 mM EDTA pH 8; 1.4 M NaCl; 0.5% beta‐mercaptoethanol), followed by amplification and sequencing of partial regions of the large ribosomal subunit (LSU) of rRNA gene, that is, D1–D3 and D8–D10, the internal transcribed spacer regions and 5.8S rRNA gene (ITS/5.8S) as described in Verma, Hoppenrath, Dorantes‐Aranda, et al. (2016) (see Table S1 for details).

Maximum‐likelihood (ML) and Bayesian inference (BI) analyses on individual alignments of ITS/5.8S, D1–D3, and D8–D10 rDNA LSU regions were performed in MEGA v6 (Tamura, Stecher, Peterson, Filipski, & Kumar, 2013) and Geneious v6 (Kearse et al., 2012), respectively, as described in Verma, Hoppenrath, Dorantes‐Aranda, et al. (2016). In addition, concatenated sequence for each strain was prepared using GBLOCKS (Castresana, 2000). Multiple sequence alignments were performed in MEGA v6 using ClustalW v1.6 (Thompson, Higgins, & Gibson, 1994) with the closely related *O*. cf. *ovata* HER27 and *O. rhodesae* strains HER6 and HER20 as outgroups. ML trees were produced using Tamura‐Nei (T92) + 5 gamma (G) category substitution model with 1,000 bootstrap replications. Bayesian analysis was performed in Geneious v6 using MrBayes v3.2 (Ronquist & Huelsenbeck, 2003) with general time reversible + G model. Four independent Markov chain Monte Carlo simulations were run simultaneously for 2 × 10^6^ generations and sampled every 1,000 generations with the first 1,000 trees discarded as burn‐in.

Molecular diversity indexes (pairwise uncorrected *p*‐distance) between and within sites were estimated from individual and concatenated sequence datasets using the *p*‐distance model (1,000 bootstrap replicates) in MEGA v6. All positions containing gaps and missing data were eliminated for the analyses. Mean number pairwise differences, nucleotide diversity (*π*), no. of polymorphic sites (*S*), and haplotype diversity (*H*_d_) for each site were calculated with concatenated sequences using Arlequin v3.5 (Excoffier, Laval, & Schneider, 2005). A statistical parsimony network was obtained with TCS v1.18 using default settings (Clement, Posada, & Crandall, 2000).

### 2.3 Growth and cell volume analysis

Fifty‐three clonal strains were selected to assess variability in the selected phenotypic characteristics. Strains were maintained for one year in the culturing conditions described above prior to measuring growth rates. Growth rates (*µ*) were determined during exponential growth (8–10 days of subculturing) using a FastOcean (Chelsea Technologies Group) Fast Repetition Rate Fluorometry (FRRf) measurements as described in Suggett et al. (2015), from at least three sequential dilutions. Fluorescence measurements were periodically verified against independent cell counts via microscopy as described in Hennige et al. (2009).

Cell dimensions during exponential growth were measured under 200× magnification using a calibrated eyepiece attached to an inverted microscope (Eclipse TS100, Nikon) equipped with brightfield optics. Samples were fixed in 1% Lugol's solution for imaging, and measurements of dorso‐ventral diameter (DV), trans‐diameter width (*W*), and anterior–posterior diameter (AP) were performed using ImageJ v1.48 (Abràmoff, Magalhães, & Ram, 2004; *n* = 20 per strain). Calculations of cell volume were performed assuming ellipsoid cell shape using equation of Sun and Liu (2003):

(1) V=π/6DV·W·AP

### 2.4 Toxin analysis via LC‐MS/MS

*Ostreopsis* cultures were harvested during late‐stationary phase (Day 19–21) via centrifugation (50 ml; 2,300 *g*; 10 min; room temperature) for screening PLTX‐like compounds using a quantitative LC‐MS/MS method at the Cawthron Institute, New Zealand, as described in Selwood et al. (2012). Briefly, this analytical approach monitors substructures generated by oxidative cleavage, using periodic acid, of vicinal diol groups present in intact toxins. This yields an amino‐aldehyde common to known PLTX‐like compounds, used for quantification, and an amide‐aldehyde that varies depending on the toxin analogue. A commercially available PLTX standard was used to generate a calibration curve and enable unambiguous identification of the oxidation products. The limit of detection (LoD) was determined as 0.5 ng/ml for the PLTX amine fragment. The relative standard deviation of repeatability for LC‐MS of oxidized PLTX standards was <10% and <8% for amino aldehyde and amide aldehyde, respectively, at 1 or 2 ng/ml, making this method suitable for monitoring trace levels of PLTX‐like compounds (Selwood et al., 2012).

### 2.5 FRRf photophysiology

Samples were acclimated to low light (*c*. 5 μmol photons m^−2^ s^−1^) for 20 min to relax nonphotochemical quenching (NPQ) and minimize chlororespiratory effects (Hill & Ralph, 2008; Kromkamp & Forster, 2003). Aliquots of 3 ml were loaded into borosilicate test tubes for FRRf measurements and temperature‐controlled via continuous circulation of water through the FRRf optical head via a heater–cooler circulator (SC100; Thermo Fisher Scientific Inc.). The FRRf was programmed to deliver single‐turnover induction of Photosystem II (PSII) via sequences consisting of 100 flashlets of 1 μs spaced 2 μs apart. Measurements were averaged from 40 consecutive sequences, with 150‐ms intervals between each sequence (Hughes, Campbell, et al., 2018; Hughes, Varkey, et al., 2018). Fluorescence transients were fitted to the biophysical model of Kolber, Prášil, and Falkowski (1998) using FastPro8 software v1.0.55 (Chelsea Technologies, UK) to retrieve minimum (*F*_o_, *F*′) and maximum (*F*_m_, *F*_m_′) PSII fluorescence (instrument units), PSII functional absorption cross section, σ_PSII_^(ʹ)^ (nm^2^ PSII^−1^), and PSII photochemical efficiency ϕ_PSII_^(ʹ)^ (unitless), where prime notations denote measurements performed in the light‐acclimated state (Hughes, Campbell, et al., 2018). Background fluorescence was measured on 0.2µm syringe‐filtered samples and subtracted from FRRf measurements.

### 2.6 FRRf photosynthetic‐irradiance response

Photosynthesis‐irradiance (PE) response was assessed using triplicate fluorescence light curves. The FRRf was programmed to deliver 15 steps of increasing irradiance from 0 to 1,208 μmol photons m^−2^ s^−1^, with each step lasting 20 s, thus reflecting a “Rapid Light Curve” (RLC) protocol (Suggett et al., 2015). Cell‐normalized rates of electron transport through PSII (ETR_PSII_, µmol e^−^ cell^−1^ hr^−1^) at each light step were calculated as:

(2) $${\text{ETR}}_{\text{PSII}}=E\cdot \sigma_{\text{PSII}}^{\prime }\cdot \left[\text{RCII}\right]\cdot qP\cdot \Phi_{\text{RCII}}\cdot {\left[\text{cells}\;m^{-3}\right]}^{-1}\cdot C$$

where *E* is irradiance (µmol photons m^−2^ s^−1^), [RCII] is the concentration of PSII reaction centers (mol RCII m^−3^), and *qP* (where *qP* = [*F*_m_′–*F*′]/[*F*_m_′–*F*_o_′]) is an estimate of the fraction of “open” [RCII] (Suggett, Moore, Hickman, & Geider, 2009). Ф_RCII_ describes the quantum yield of [RCII] and is assumed to be a constant of 1 mol electron (mol photon)^−1^, [cells/m^3^] is the mean cell count of the sample, and *C* is a factor converting σ_PSII_^ʹ^ from units of nm^2^ PSII^−1^ to m^2^ mol PSII^−1^_,_ and seconds to hours (=2.168 × 10^9^). [RCII] was estimated fluorometrically using the algorithm of Oxborough et al. (2012) as:

(3) $$\left[\text{RCII}\right]=K_{a}\cdot \frac{F_{o}}{\sigma_{\text{PSII}}},$$

where $K_{a}$ represents an instrument‐specific constant (m^−1^). PE curves were fitted to the hyperbolic tangent function of Platt, Gallegos, and Harrison, (1981), using MS Solver to derive the maximum photosynthetic rate (ETR_max_), light utilization efficiency (*α*), and light saturation parameter (*E_k_*).

To compare the transient influence of photochemical quenching (1–*C*) versus non‐photochemical quenching (NPQ) in governing photosynthetic efficiency across strains, we followed the procedure of Suggett et al. (2015) and calculated NPQ as ([*F*_v_′/*F*_m_′]/[*F*_v_/*F*_m_]), hereafter referred to as (1–*Q*). Importantly, while the RLC is a powerful approach to probe photosynthetic response, its relatively short length (20 s) provides limited time for the generation of NPQ mechanisms; and thus, in this study, values of (1–*Q*) are most useful to identify strains capable of rapid upregulation of non‐photochemical energy dissipation (Lavaud, Strzepek, & Kroth, 2007).

### 2.7 Pigments and photosynthetic unit size

Fifteen milliliters of culture was filtered on GF/F filters, immediately resuspended in 3 ml of acetone (90%, *v*/*v*) and stored overnight at 4°C to allow for pigment extraction. Absorbance of extracts was measured at 665 and 750 nm (UV/VIS, JASCO 7800) to determine chlorophyll‐*a* (Chl‐*a*) concentration using the following equation (Ritchie, 2006):

(4) $$\text{Chl}\;-\;a\;\left(\mu g/L\right)=\left(11.4062\cdot \left[A_{665}-A_{750}\right]\cdot v\cdot 10^{3}\right)/\left(c_{o}\cdot V\right)$$

where *A*_665_ = blank‐corrected absorbance at 665 nm, *A*_750_ = blank‐corrected absorbance at 750 nm, *v* = volume of acetone solution used for extraction (ml), *c*_o_ = cell path length (cm), and *V* = filtered sample volume (ml). Size of the photosynthetic unit (PSU) was calculated from Chl‐*a* concentration and [RCII] measures as per Hughes et al. (2020):

(5) $$\text{PSU}\;\text{Size}\;(\text{mol}\;\text{Chl}\;-\;a\;{\left[\text{mol}\;\text{RCII}\right]}^{-1})=\text{Chl}\;-\;a/\left[\text{RCII}\right]$$

### 2.8 Statistical analysis

One‐way analysis of variance (ANOVA) and Tukey's HSD post hoc tests were applied to identify differences across strains from each site for *µ*, total cell volume, *F*_v_/*F*_m_, *σ*_PSII_, ETR_max_, *α*, and *E*_k_. Analyses were conducted using SPSS v24 (IBM Corp.). Correlation of certain phenotypic traits was determined using the Pearson correlation coefficient (*r*) using SigmaPlot v12 (Systat Software, San Jose, CA). When data failed to meet assumptions of normality, the nonparametric Kruskal–Wallis test was applied. To identify various phenotypic functional groupings among strains based on light harvesting and utilization parameters (*σ*, *F*_v_/*F*_m_, [RCII] cell^–1^, (1–*C*), and (1–*Q*), together with *µ*, cell volume and quantity of PLTX‐like compounds, cluster analysis, and multidimensional scaling (MDS) were performed using PRIMER‐E v6.1 (PRIMER‐E Ltd.). Values of fluorescence ratios, that is, *F*_v_/*F*_m_, (1–*C*) and (1–*Q*), were arcsine transformed to stabilize variance for MDS analysis. Prior to analysis, variables were standardized to account for order of magnitude differences in value ranges. The correlation matrix was used for a two‐dimensional principal component analysis (PCoA), and principal components were displayed as an ordination plot.

## 3 RESULTS

### 3.1 Phylogeographic structures and genetic diversity

**FIGURE 3:**
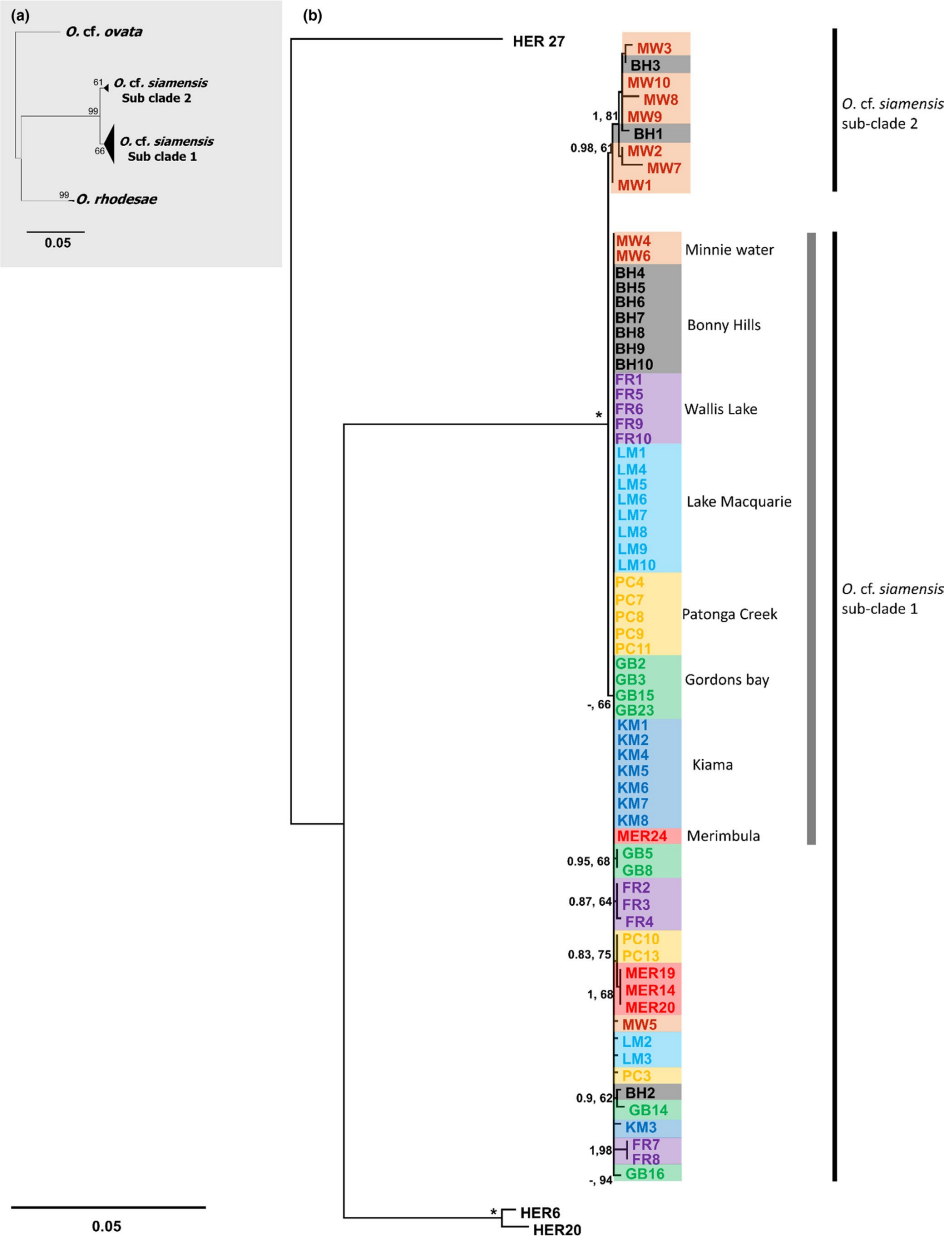
Phylogenetic analysis of *Ostreopsis* cf. *siamensis* strains**.** (a) Maximum‐likelihood (ML) tree based on concatenated ITS/5.8S, D1‐D3, and D8‐D10 regions of the LSU rRNA gene representing the two subclades of *Ostreopsis* cf. *siamensis*. (b) Phylogram representing the various haplotypes within the two subclades. The internal grey line represents haplotype 1 common to all locations. Numbers at nodes represent posterior probabilities from Bayesian Inferences (BI) and bootstrap support values from ML based on 1,000 pseudoreplicates. Only bootstrap values >50% are shown. * Represents 1, 100 support values for BI and ML, respectively. Color codes represent origin of strains as represented in Figure 2

**FIGURE 4:**
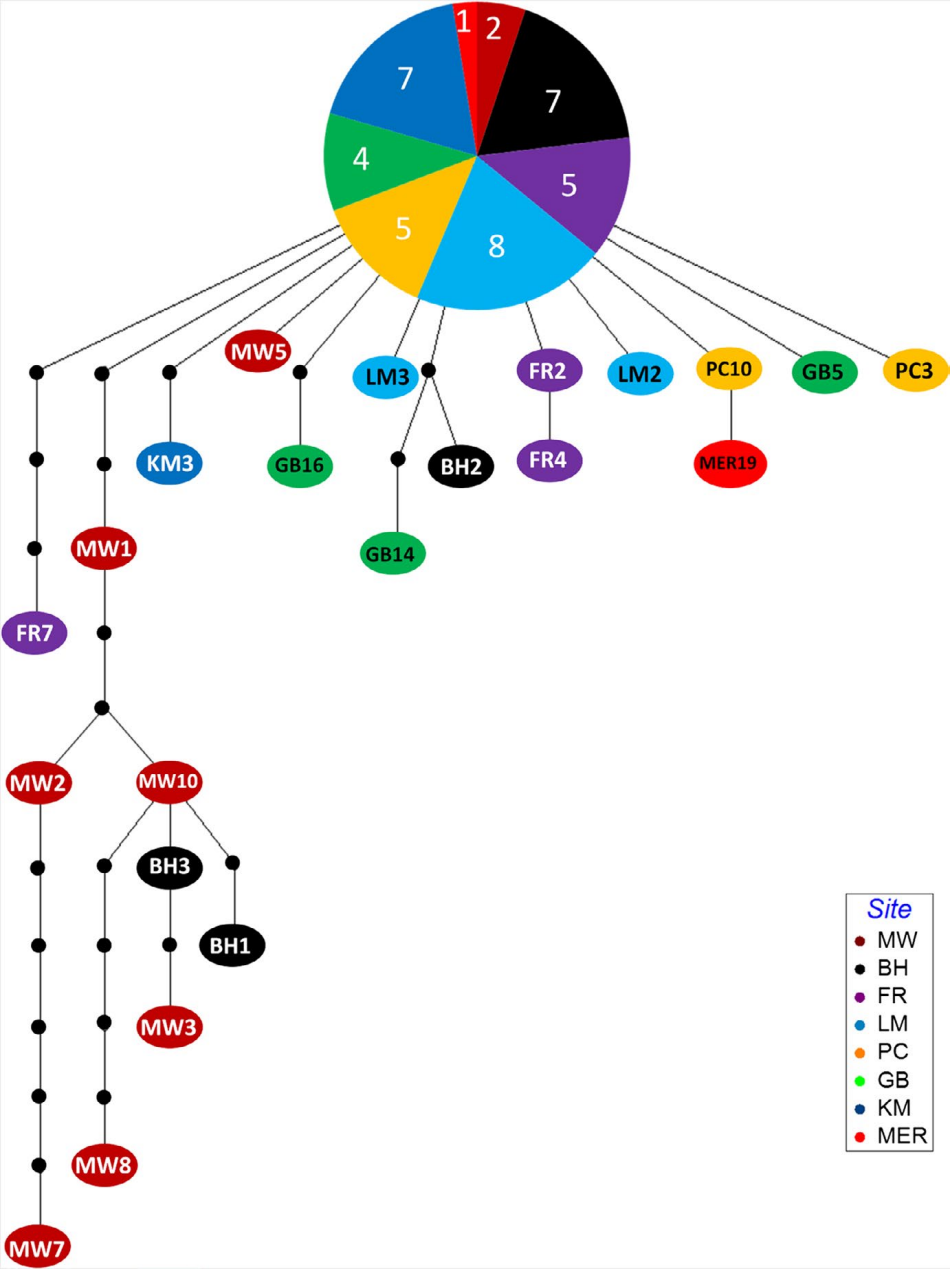
Haplotype network based on 68 concatenated sequences using statistical parsimony. Mutation steps are shown in black dots. Color codes represent origin of strain as represented in Figure 2. Strain codes are representatives of haplotypes as described in Table 1. Numbers in the pie chart represent the number of isolates from each location that belongs to the common haplotype (Haplotype 1)

The monophyly of 68 *O*. cf. *siamensis* strains was strongly supported by both ML and BI phylogenetic analyses for all three molecular markers (Table 1, Figure S1A–C). Phylogenetic analysis of concatenated data was broadly congruent with the analyses based on individual genes, yielding two distinct subclades (Figure 3) and 23 haplotypes (8 in subclade 2 and 15 in subclade 1; Figure 4). Only strains from the northernmost sampling sites, that is, Minnie Water (MW1, MW2, MW3, MW7, MW8, MW9, and MW10) and Bonny Hills (BH1 and BH3) clustered to form subclade 2, whereas all other strains clustered in subclade 1 (Figure 3). Unique haplotypes were identified at all sampling sites, with all geographic populations sharing a common haplotype (hereafter referred to as haplotype 1; Figure 4; Table 1). A total of eight haplotypes were identified in Minnie Water (MW), four each in Bonny Hills (BH), Forster (FR) and Gordons Bay (GB), three each in Lake Macquarie (LM) and Patonga Creek (PC), and only two in Kiama (KM) and Merimbula (MER) strains, respectively (Figure 4, Table 1). Uncorrected *p*‐distances indicated higher molecular diversity within the northern sampling sites, that is, MW, BH, and FR (0.002–0.005; Table S3A), with genetic distances between strains from MW and other sampling sites being greater in comparison with differences observed between strains from other sites (0.005–0.006; Table S3B).

### 3.2 Growth rates, cell volume, and toxin production

**FIGURE 5:**
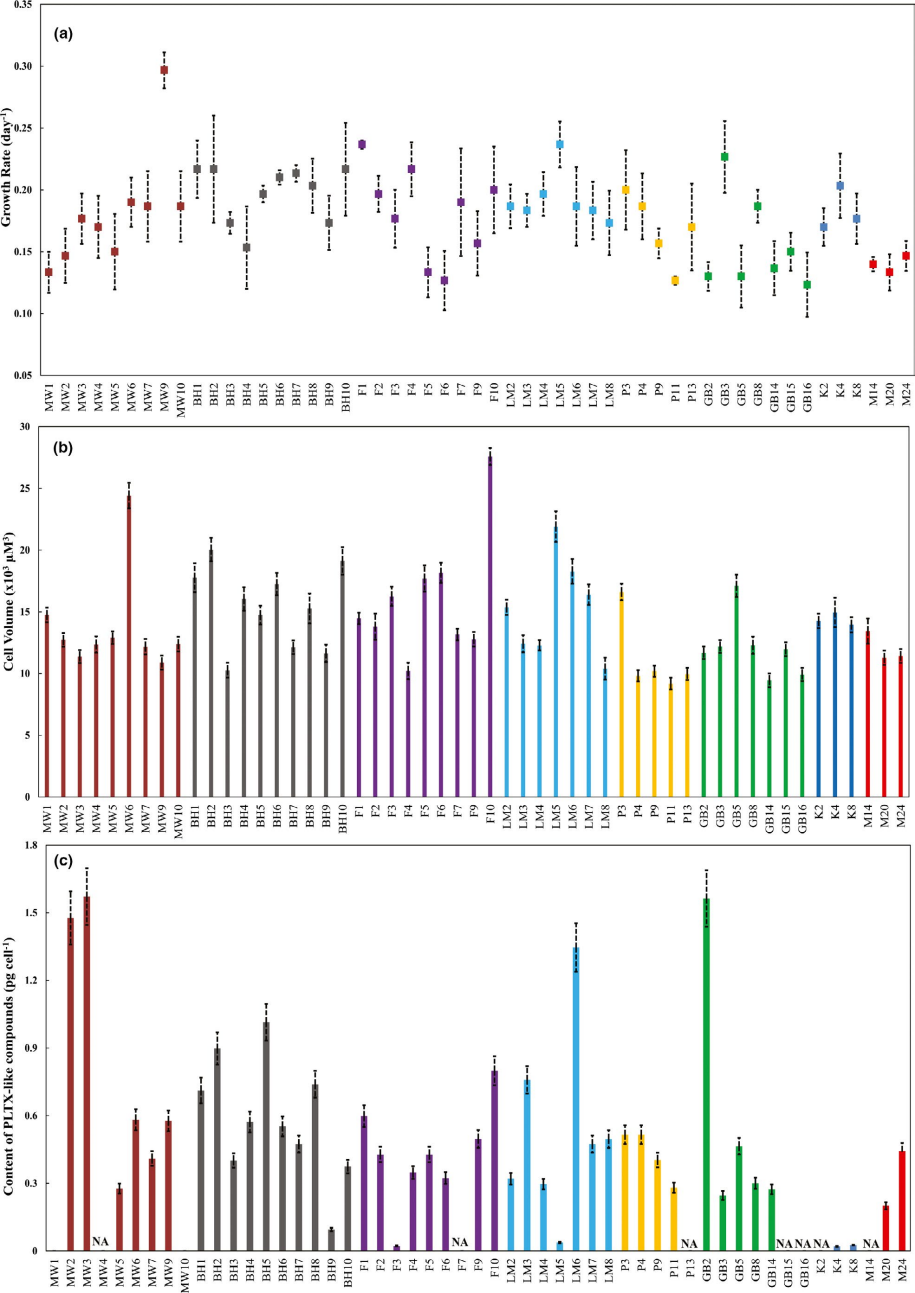
Phenotypic variation of 53 *Ostreopsis* cf. *siamensis* strains. (a) Mean growth rates. Error bars represent standard error of three replicate measurements. (b) Cell volume. Error bars represent standard error of 20 measurements. (c) Cellular content of PLTX‐like compounds. Error bars represent 8%–10% relative standard deviation of repeatability for LC‐MS measurements. NA represents toxin amount below the limit of detection. Color codes represent origin of strain as represented in Figure 2

Mean growth rates (*µ*) ranged from 0.12 ± 0.03 (GB16) to 0.27 ± 0.04 d^−1^ (MW9) among the 53 strains (mean: 0.18 ± 0.004 d^−1^) in this study with significant differences among strains isolated from different locations (ANOVA, *F* = 2.945; *p* < .01; Figure 5a, Tables S4A and S5A). Significant differences in *µ* among strains from the same sampling site were also identified but in strains isolated from MW and GB (ANOVA, *F* = 4.093, 3.213, respectively; *p* < .05; Table S5B–I). The coefficient of variation ranged from 30.9% in MW to 12.9% in MER (Table S5J).

Cell volumes ranged from 9.17 ± 0.47 × 10^3^ (PC4) to 2.76 ± 0.69 × 10^4^ µm^3^ (FR10) with significant differences among strains isolated from different sampling sites (ANOVA, *F* = 18.52, *p* < .01; Figure 5b, Tables S4A and S5A). The mean cell volume of *O*. cf. *siamensis* strains investigated in this study was 1.4 ± 0.52 × 10^4^ µm^3^, and varied significantly among strains isolated from the same sampling site with the exception of those isolated from KM and MER (ANOVA, *p* > .05; Tables S4A and S5B‐I). The coefficient of variation ranged from 36.2% in FR to 26% in KM (Table S5J). The cell volumes did not correlate with growth rate of the strains (correlation coefficient (*r*) = .27; *p* > .05; Table S5K).

LC‐MS/MS analysis of the oxidized cellular extracts yielded diverse qualitative and quantitative trends among the investigated strains (Figure 5c and Table S4A). The amino‐aldehyde fragment, common to all known PLTX, ovatoxin, and ostreocin analogues, as well as an amide‐aldehyde fragment was detected from 18 strains, thereby confirming the presence of PLTX‐like compounds in those cultured extracts (Figure S2A). Among 26 strains, only the amino‐aldehyde fragment, but no amide‐aldehyde fragment, could be detected (Figure S2B). This suggests the presence of structurally related PLTX‐like compounds, albeit with some structural differences from PLTX in the amide‐aldehyde portion of the molecule. Strains MW1 and MW10, isolated from Minnie water, displayed a unique LC‐MS/MS profile, where only the amide‐aldehyde fragment was observed and not the common PLTX amine‐aldehyde fragment (Figure S2C), due to which their toxin content could not be quantified. Such toxin profiles are indicative of the production of potentially uncharacterized PLTX‐like compounds by these strains which require further structural elucidation. The amounts of PLTX‐like compounds were found below the detection limit for 7 strains (Figure 5c and Figure S2D). These “nontoxic” strains were observed from all sampling sites, with the exemptions of BH and LM latitudinal populations. The amount of PLTX‐like compounds detected from the *O*. cf. *siamensis* strains did not correlate with their corresponding cell volumes (*r* = .15; *p* > .05) but showed a weak, yet significant correlation to growth rates (*r* = .33; *p* < .05; Table S5K).

### 3.3 Photobiology

**FIGURE 6:**
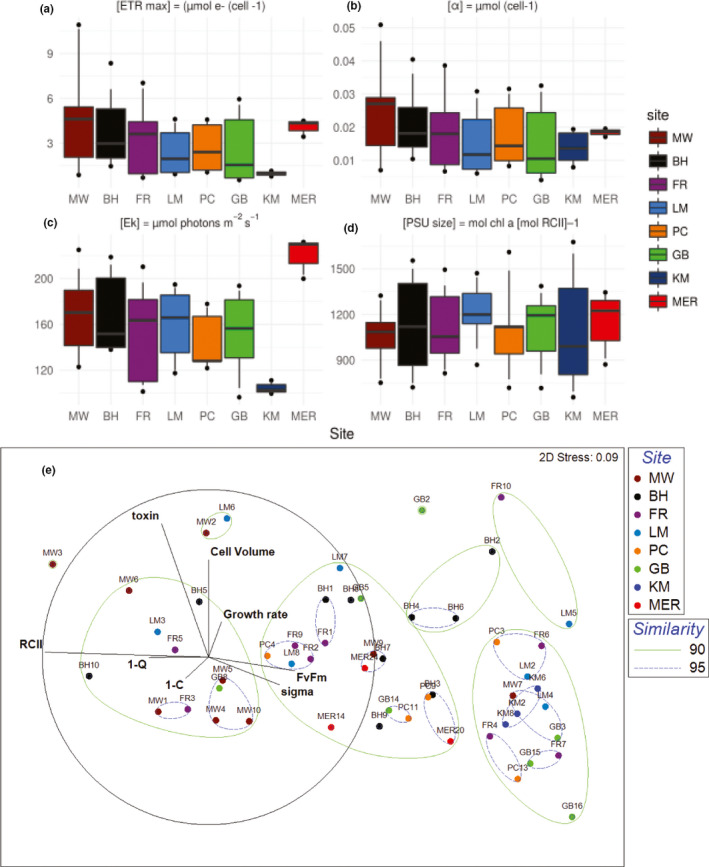
Functional groupings based on phenotypic variability in *Ostreopsis* cf. *siamensis* strains. Whisker plots of (a) maximum photosynthetic rate (ETR_max_); (b) light utilization efficiency (*α*); (c) light saturation parameter (*E_k_*); (d) photosynthetic unit (PSU) size among *Ostreopsis* cf. *siamensis* isolates based upon sampling sites. Whiskers above and below the boxes indicate the 90/10 percentiles, dots the respective 95/5 percentiles. (e) Cluster analysis and multidimensional scaling (MDS) plot of the average of each variable, including light harvesting, that is *F*_v_/*F*_m_ *,* σ_PSII_, cellular [RCII] concentration, light utilization, that is, (1−*C*) and (1−*Q*), along with growth rates, cell volume, and amounts of PLTX‐like compounds per variant. Similarity is shown at the 90% and 95% levels, and vectors driving the clustering are shown in black

After being maintained in identical growth conditions for over a year, *O*. cf. *siamensis* strains exhibited significant variability in photophysiological parameters governing both dark acclimated and irradiance responses. ETR_max_ ranged from 0.49 ± 0.02 (GB3)–11.19 ± 1.5 µmol e^−^ cell^−1^ hr^−1^ (MW3; mean: 3.36 ± 0.39 µmol e^−^ cell^−1^ hr^−1^) among the 53 strains and varied significantly among isolates from each location with the exception of MER (Figure 6a and Table S5A–I). A similar trend was observed for *α*, with values ranging from 4 ± 0.1 × 10^–3^ (GB16) – 5.6 ± 0.7 × 10^–2^ µmol e^−^ cell^−1^ hr^−1^ (MW3; mean: 1.9 ± 0.1 × 10^–2^ µmol e^−^ cell^−1^ hr^−1^; Figure 6b and Table S5A–I). Interestingly, the light saturation parameter (*E_k_*), that is, the irradiance at which photosynthesis becomes light‐saturated, ranged from 90 to 240 μmol photons m^−2^ s^−1^ among isolates, despite cultures being maintained under a growth irradiance of 60 ± 15 µmol photons m^−2^ s^−1^ (Figure 6c). Significant variation in *E_k_* was observed among isolates from the same geographic population, with the exceptions of KM and MER (Table S5B–I). This potentially suggests that clones isolated from northern and mid‐latitudinal populations along the EAC coastal regions exhibit more diverse light utilization and harvesting strategies; however, the strains established from the southern sampling sites are relatively more homogenous in their photophysiological strategies (Tables S4B and S5B–I).

*F*_v_/*F*_m_ ranged from 0.42 ± 0.01 (MW2) to 0.59 ± 0.005 (GB3; mean: 0.5 ± 0.01), thus falling within the expected range for dinoflagellate cultures maintained under steady‐state growth (Suggett et al., 2015; Table S4C). σ_PSII_ typically varied from 3.08 ± 0.08 (LM7) to 3.97 ± 0.17 nm^2^ PSII^−1^ (MW5; mean: 3.5 ± 0.05 nm^2^ PSII^−1^; Table S4C); yet, unlike previous studies of other dinoflagellates, for example, Suggett et al. (2009, 2015), did not exhibit a significant inverse correlation with *F*_v_/*F*_m_ (*r* = .19; *p* > .05). Chl‐*a* concentration varied from 30 ± 2 (MW7) to 105 ± 3 pg per cell (MW5) among *Ostreopsis* isolates (mean: 60 ± 3 pg Chl‐*a* cell^−1^) which are similar values to those reported from other *Ostreopsis* species (Pezzolesi et al., 2014; Table S4C). When normalized to cell density, [RCII] ranged from 28 ± 20 (GB16) to 426 ± 28 mol [RCII] m^−3^ × 10^–18^ cell^−1^ (BH10; mean: 162 ± 43 mol [RCII] m^−3^ × 10^–18^ cell^−1^), while normalization to cellular Chl‐*a* concentration yielded a PSU size range of 482 ± 32 (MW7)–1,850 ± 40 mol Chl‐*a* (mol [RCII])^−1^ (GB8) (mean: 1,044 ± 43 mol Chl‐*a* (mol [RCII])^−1^) and neither appeared to show an obvious pattern linked to geographic origin (Figure 6d and Table S4C).

The MDS analysis based on photoacclimation (*F*_v_/*F*_m_, *σ*_PSII_ and [RCII cell^−1^]) and light utilization, that is, (1–*C*) and (1–*Q*), is presented in Figure S3. Light utilization parameters were measured at 400 µmol photons m^−2^ s^−1^, representing a saturating irradiance level for all strains. The variability among functional groups was predominately driven by cellular [RCII] concentration and *σ*_PSII_ (i.e., light harvesting parameters; similarity matrix cutoff: 90% and 95%; Figure S3). This indicates that *O*. cf. *siamensis* strains may employ several distinct adaptive strategies to optimize photosynthetic capacity relative to metabolic demand and microenvironments including (a) modifying the size of the PSII antenna complex serving the core reaction center (σ‐type strategy); (b) increasing the number of PSII units (*n*‐strategy); or (c) a combination of both.

The MDS plot of 53 *O*. cf. *siamensis* strains based on light harvesting and utilization parameters, *µ*, cell volume and amounts of PLTX‐like compounds (denoted as toxin) is represented in Figure 6e. Phenotypic differences based on location were not evident as strains isolated from the same latitudinal population spread across different phenotypic “clusters” (similarity matrix cutoff: 90% and 95%). The ordination plot with respect to two principal component axes (PC I and PC II) based on PCoA analysis of eight aforementioned eco‐physiological traits is shown in Figure S4. PC I and II account for 76% and 12% of the total variation, respectively (Table S6A). Axis 1 has strong positive loadings for [RCII] and strong negative loading for *σ*_PSII_ (Table S6B). This highlights variable adaptive light‐harvesting strategies among the strains. Axis 2 has strong positive loadings for PLTX‐like compounds produced by the strains with negative loadings for photophysiological variables. This potentially reflects a trade‐off in photophysiological fitness to invest in producing energetically costly PLTX‐like compounds.

## 4 DISCUSSION

The nature and extent of intra‐ and interpopulation clonal variations are important concepts for understanding population dynamics of microbial eukaryotes (Alpermann, Tillmann, Beszteri, Cembella, & John, 2010; Brandenburg et al., 2018; Kremp et al., 2012; Tillmann, Alpermann, da Purificação, Krock, & Cembella, 2009). In this study, we report a multitude of coexisting phenotypic characteristics in a subset of *O*. cf*. siamensis* strains isolated from eight geographic populations along the coastal regions of the EAC, suggestive of a broad capacity to acclimate to a wide range of environmental conditions in highly variable habitats. This study is the first to reveal high genetic and phenotypic variability between and within populations of a benthic harmful algal species. Fifty‐three *O*. cf. *siamensis* strains examined in this study had varying growth‐, morphological‐, toxin‐, and photophysiological properties. Variability in the investigated phenotypic traits is independent of the clustering of strains into distinct genotypic subclades, but more significant among strains from the northern geographic populations, similar to the trend observed with the genetic data, suggestive of a more diverse and adaptive population in the northern subtropical regions of the EAC with a larger number of “eco‐” and “geno‐“ types”. Such observations in phenotypic and genotypic variability suggest that genetic diversity is significantly linked to increased diversity in phenotypic responses in microbial populations as described in Ackermann (2015). As phenotypic diversity is crucial in buffering population responses to environmental variation, understanding its role in benthic species, capable of causing toxic blooms, along climate change “hot‐spot” will not only improve our understanding of harmful algal bloom formation along the warming EAC, but will also facilitate our knowledge of marine microbial eukaryotic populations in the current global environmental change (Brandenburg et al., 2018; Driscoll et al., 2016).

### 4.1 Phylogeography and population divergence

Dispersal mechanisms and their impact on population differentiation in benthic marine microbial eukaryotes, as compared to planktonic species, are not well understood. Some studies have shown that passive transport of vegetative and/or resting cysts on floating and drifting objects such as macrophyte “rafts” and plastic debris can occur (Larsson, Laczka, Suthers, Ajani, & Doblin, 2018; Masó, Garcés, Pagès, & Camp, 2003; Penna et al., 2010). However, whether such transport occurs sufficiently frequently to be associated with the level of gene flow is not known.

In this study, *O*. cf. *siamensis* populations consisted of a tight assemblage of haplotypes separated by a small number of mutations (Figures 3 and 4). The concatenated alignment used in this study allowed for a more strongly supported topology (Murray, Jørgensen, Ho, Patterson, & Jermiin, 2005), than is often found in phylogenies of *Ostreopsis*, which divided the *O*. cf. *siamensis* into two subclades, with the new subclade comprising of certain strains isolated from sampling sites MW and BH. This suggests that *O*. cf. s*iamensis* “species‐complex” could possibly exist as a tight assemblage of closely related genotypes but further taxonomic investigation is required to delineate this possibility. We noticed a larger number of polymorphic sites in the LSU D1‐D3 region, compared to other rDNA regions, similar to phylogeographic surveys of other microbial eukaryotic genera (Auwera & Wachter, 1998; Godhe, McQuoid, Karunasagar, Karunasagar, & Rehnstam‐Holm, 2006; Sato et al., 2011; Table S3A and B). “Haplotype 1” was found to be abundant and geographically widespread among all sampling site, but a large number of rare haplotypes were reported from the northern and mid‐latitude sites of the EAC, that is, MW, BH, FR, and GB (Figure 4). This observation suggests that these sites consisted of populations with larger amounts of genetic diversity, but such a pattern is not clear in populations further south. One possible explanation for this diversity pattern is that the southerly populations may have colonized their habitats more recently. It is well‐documented that the EAC has increased its southward extension over the past 50 years, and this extension has been associated with range expansions in species of invertebrates, zooplankton, coastal estuarine fish, and pelagic harmful algal species along the southeast coastline of Australia (Johnson et al., 2011; Last et al., 2011; Ling, Johnson, Ridgway, Hobday, & Haddon, 2009; McLeod, Hallegraeff, Hosie, & Richardson, 2012).

### 4.2 Intraspecific trait variation

#### 4.2.1 Growth rates and cell volume

Growth rate (*µ*) is a significant variable in population ecology, as it integrates numerous biochemical processes to yield a single “output” that reflects the ability of individuals to adapt to the changing environments (Alpermann et al., 2010; Tillmann et al., 2009). However, this depends on a wide range of interplays between intrinsic (genetic/epigenetic) and environmental factors. All investigated strains in our study were acclimated under identical growth conditions for more than a year prior to experimentation, yet measured growth rates varied from 0.12 to 0.27 d^−1^. Such variation is relatively large compared to previous interstrain and/or interclade dinoflagellate studies (Kremp et al., 2012, 2016; Suggett et al., 2015; Tillmann et al., 2009; Figure 5a).

Cell size is often considered a “master trait” to predict light harvesting and utilization as well as nutrient acquisition in microalgae (Harvey, Menden‐Deuer, & Rynearson, 2015; Suggett et al., 2015; Wu, Campbell, Irwin, Suggett, & Finkel, 2014). Variability in growth rates due to cell volume are known to be driven by metabolic processes such as nutrient uptake, assimilation, carbon fixation, and respiration (Litchman & Klausmeier, 2008; Litchman et al., 2007). Cell volumes reported in our study ranged from 9.2 × 10^3^ to 2.7 × 10^4^ µm^3^. Significant variations in cell volumes between “large‐” and “small‐” sized strains were reported from each sampling site, with the exception of southern locations (Figure 5b). No significant morphological trait among strains from subclade 2 was identified during the volumetric analysis performed using light microscopy. However, the presence of cryptic differences between the two subclades needs to be verified through thorough microscopic investigation. Previous studies have also reported variability in cell volumes among *O*. cf. *ovata* strains, but not to such an extent (Pezzolesi et al., 2014). Size‐scaling relationships from other microbial eukaryotic studies have suggested that small cells are unlikely to prevail in bloom‐forming conditions because of constraints on nutrient uptake and reduced biosynthetic abilities, whereas large cells are limited by the conversion of nutrients into biomass due to size‐related constraints levied on intracellular resource transportation (Marañón, 2015; Ward, Marañón, Sauterey, Rault, & Claessen, 2017). The resulting trade‐off between these opposing size‐driven limiting processes could potentially explain the varying sizes among *Ostreopsis* strains from different, as well as, the same sampling locations in this study. The traditional model of estimating µ in phytoplankton suggests that µ decreases with increasing cell size (Eppley & Sloan, 1966). However, in our study no significant correlation of physiological parameters could be established with cell volume. For the clonal populations studied, the stages of population development and cell cycle are not known, but future studies on these phenotypic traits can benefit from incorporating such information into the experimental design.

#### 4.2.2 Toxicity

The amounts of PLTX‐like compounds detected in this study varied substantially among strains within and between sites (Figure 5c and Figure S2A–D). Variation in cellular toxin content and composition within local populations has been reported with paralytic shellfish toxins in *Alexandrium* spp. and karlotoxins in *Karlodinium veneficum* (Alpermann et al., 2010; Bachvaroff, Adolf, & Place, 2009; Kremp et al., 2016; Tillmann et al., 2009). However, in these cases, either only a few strains were examined, or strains were isolated from widely different geographic regions. In this study, while many strains produced PLTX‐like compounds at comparable cellular concentrations to previous *Ostreopsis* reports from the EAC region (Verma, Hoppenrath, Dorantes‐Aranda, et al., 2016; Verma, Hoppenrath, Harwood, et al., 2016), some strains were apparently “nontoxic”, and others produced a potentially unique toxin analogue (e.g., MW1 and MW10). Numerous PLTX analogues (such as ostreocin‐A, ostreocin‐B, ostreocin‐D, ostreocin‐E1, ovatoxins a‐k, mascernotoxin‐A, mascernotoxin‐ B, mascernotoxin‐C, and isobaric palytoxin) have been described from several *Ostreopsis* species to date (Ciminiello, Dell'Aversano, Dello Iacovo, et al., 2012; Ciminiello, Dell'Aversano, Iacovo, et al., 2012; Dell'Aversano et al., 2014; Tartaglione et al., 2016; Terajima, Uchida, Abe, & Yasumoto, 2018, 2019). However, the LC/MS‐MS approach used in our study only monitors substructures generated by the oxidative cleavage of large PLTX‐like compounds and does not illustrate the entire diversity of potential analogues produced by a certain strain. Further studies investigating the complete structure of toxic compounds, combined with a genetic understanding of the complex toxin biosynthesis pathways, may fully illuminate the diversity of PLTX‐like compounds produced by *O*. cf. *siamensis* strains from the EAC region (Verma, Barua, et al., 2019; Verma, Kohli, Harwood, Ralph, & Murray, 2019).

John et al. (2015) have suggested that diversity in the production of secondary metabolites within a population of a HAB species might allow for intraspecific facilitation, particularly as a defense mechanism against grazers. Recent studies have reported *Ostreopsis* species as likely prey for heterotrophic dinoflagellates and grazers due to their slow swimming speed (Du Yoo et al., 2015). The induction of harmful effects on the behavior and survival of grazers when exposed to *Ostreopsis* species has been demonstrated (Neves, Contins, & Nascimento, 2018). This suggests that PLTX‐like compounds could potentially be produced as a grazer defense trait, as a trade‐off for lower motility of this species and/or also at the expense of photobiological fitness as the cost of producing such energetically costly molecules, but remains to be investigated in detail (Figure 6e and Figure S4). Such traits can be induced, yet the changes may occur too slowly to provide protection from immediate predation pressure (John et al., 2015). Rapid selection of highly toxic strains from a pool of individuals with varying defense capabilities might prove a more effective mechanism for populations to cope with different grazing regimes (Driscoll et al., 2016; Selander, Thor, Toth, & Pavia, 2006). Within structurally diverse populations of closely related individuals, cooperative traits can be favored as being for the “public good” and enable the success of the entire population (Hamilton, 1964). This principle of facilitation has been shown in populations of various organisms such as antibiotic‐resistant bacterial strains and toxigenic cyanobacterial species (John et al., 2015; Lee, Molla, Cantor, & Collins, 2010; Van Gremberghe et al.., 2009). Fitness of highly toxic individuals will be higher during periods of high grazing pressure, whereas low‐ or non‐toxic clonal lineages might benefit during periods when grazing pressure is low (John et al., 2015; Kremp et al., 2016).

### 4.3 Insights into photophysiology of benthic microbial eukaryotes

In this study, we used FRRf to demonstrate the interplay of factors that govern photosynthetic electron transport rate in microalgae and highlight several photo‐acclimation strategies (Falkowski & Raven, 2013; Hennige et al., 2009; Oxborough et al., 2012; Suggett et al., 2009, 2015). We demonstrate putative existence of both σ and *n*‐type photo‐acclimatory strategies among *O*. cf. *siamensis* strains from the same, as well as different locations suggestive of a localized, short‐term “niche‐” driven adaptability of photobiological functional traits (Figure S3). More functional “eco‐types” were observed from northern locations compared to southern sampling sites (Figure 6e and Figure S3). Predominance of *n*‐type strategy has been suggested as a “generalist” strategy for microalgae subjected to varying light intensities, where modifying [RCII] concentrations over antennae size (*σ*) are beneficial under conditions likely to cause photoinhibition, such as high irradiance (Hennige et al., 2009). Under more stable light regimes, an σ‐type strategy may be incorporated to reduce energetic costs. The ability to employ either an *n*‐type or σ‐type strategy can be dictated by nutrients, where low‐nitrogen availability is more conducive to σ‐type strategy as it is less energetically costly (Six et al., 2008). In coastal environments, such as the sampling sites in this study, nutrient concentrations are often relatively higher than the open ocean; hence, it can be hypothesized that *O*. cf. *siamensis* have access to sufficient resources, and can thus afford to use a higher cost *n*‐type acclimation of photosystem contents to exploit a wider range of light availability.

We also witnessed ability to upregulate NPQ rapidly (within the timeframe of RLC) in a select few strains (Figure S3). This suggests that some strains may be able to better cope with rapid fluctuations of light on short timescales, for example, the passage of clouds, coastal turbulence, or even wave‐flickers and wave‐lensing. NPQ expression in microalgae has been linked to production of photoprotective pigments to counter effects of excess light. Indeed, it has been previously determined that photoprotective compounds (e.g., carotenoids) increase during exposure to high light and ultraviolet radiation (Patil et al., 2017). *Ostreopsis* species are known to produce extracellular polysaccharide secretions (EPS) that have been linked with their bloom formation capabilities by providing mechanical resistance to dynamic wave motion (Honsell et al., 2013; Sechet et al., 2012). As such polysaccharides theoretically represent a large sink for photosynthetically derived carbon molecules, hence the differential ability to rapidly dissipate excess absorbed light energy as NPQ may play a role in EPS production among *Ostreopsis* populations experiencing transient light stress, and warrants further targeted research.

## 5 CONCLUSION

This study reports considerable interspecific genetic divergence and functional trait variation among strains of the benthic marine microbial eukaryote, *O*. cf. *siamensis,* over a north–south gradient along the east coast of Australia, with a larger number of “eco”‐ and “geno‐” types from the northern geographic populations. Among the investigated phenotypic traits, the prevalence of different light harvesting strategies highlights a greater potential for a benthic species such as *O*. cf. *siamensis* to cope with highly dynamic light regimes. Such high degree of functional trait variability among isolates may have enabled this species to colonize new environments successfully, which could aid the potential southward range expansion, and can also facilitate its rapid proliferation under varying environmental conditions. Trait variation maintained through shifting selection pressures can contribute to the prevalence and success of protistan populations under the changing global environment, and might also facilitate in bloom formation among harmful algal species.

## CONFLICT OF INTEREST

The authors declare that they have no conflict of interest.

## AUTHOR CONTRIBUTION

**Arjun Verma:** Conceptualization (lead); Data curation (lead); Formal analysis (lead); Investigation (lead); Methodology (lead); Validation (lead); Visualization (lead); Writing‐original draft (lead); Writing‐review & editing (lead). **David J. Hughes:** Data curation (supporting); Formal analysis (supporting); Investigation (supporting); Methodology (supporting); Validation (supporting); Writing‐review & editing (supporting). **D. Tim Harwood:** Data curation (supporting); Formal analysis (supporting); Investigation (supporting); Methodology (supporting); Writing‐review & editing (supporting). **David J. Suggett:** Resources (supporting); Writing‐review & editing (supporting). **Peter J. Ralph:** Supervision (supporting); Writing‐review & editing (supporting). **Shauna A. Murray:** Conceptualization (supporting); Funding acquisition (lead); Investigation (supporting); Resources (lead); Supervision (lead); Writing‐review & editing (supporting).

## Supporting information

Figure S1

Figure S2

Figure S3

Figure S4

Table S1

Table S2

Table S3

Table S4

Table S5

Table S6

## Data Availability

The sequence data have been uploaded and are available on NCBI GenBank as represented in Table S2. ITS accession numbers: MN418248–MN418315; D1–D3 LSU accession numbers: MN418317–MN418384; D8–D10 LSU accession numbers: MN396460–MN396527.
