# Supplementary material for: Functional significance of phylogeographic structure in a toxic benthic marine microbial eukaryote over a latitudinal gradient along the East Australian Current
Source: Ecol Evol. 2020 May 21;10(13):6257–73. doi: 10.1002/ece3.6358 (PMC7381561; doi:10.1002/ece3.6358)
Supplement: Supplementary file 1 — Figure S1 [file ECE3-10-6257-s001.docx]

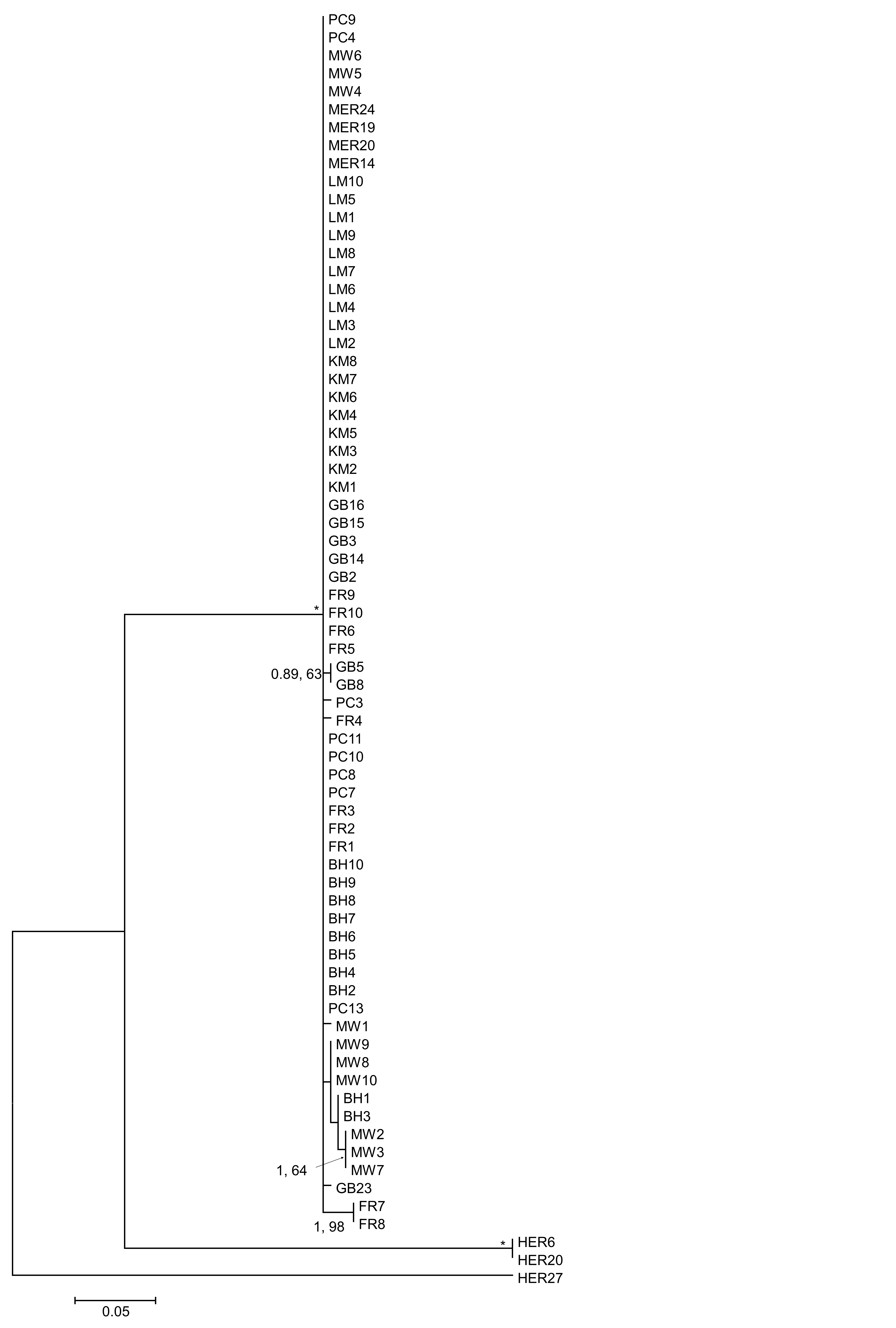


PC9

PC4

MW6

MW5

MW4

MER24

MER19

MER20

MER14

LM10

LM5

LM1

LM9

LM8

LM7

LM6

LM4

LM3

LM2

KM8

KM7

KM6

KM4

KM5

KM3

KM2

KM1

GB16

GB15

GB3

GB14

GB2

FR9

FR10

FR6

FR5

GB5

GB8

PC3

FR4

PC11

PC10

PC8

PC7

FR3

FR2

FR1

BH10

BH9

BH8

BH7

BH6

BH5

BH4

BH2

PC13

MW1

MW9

MW8

MW10

BH1

BH3

MW2

MW3

MW7

GB23

FR7

FR8

HER6

HER20

HER27

*

1, 98

1, 64

0.89, 63

*

0.05

Supplementary Fig. S1A: Maximum Likelihood (ML) phylogenetic tree of *Ostreopsis* cf. *siamensis* strains based on ITS1-5.8S-ITS2 region. Numbers at nodes represent posterior probabilities from Bayesian Inferences (BI) and bootstrap support values from ML based on 1000 pseudo-replicates. Only bootstrap values > 50% are shown.

* represents 1, 100 support values for BI and ML respectively.


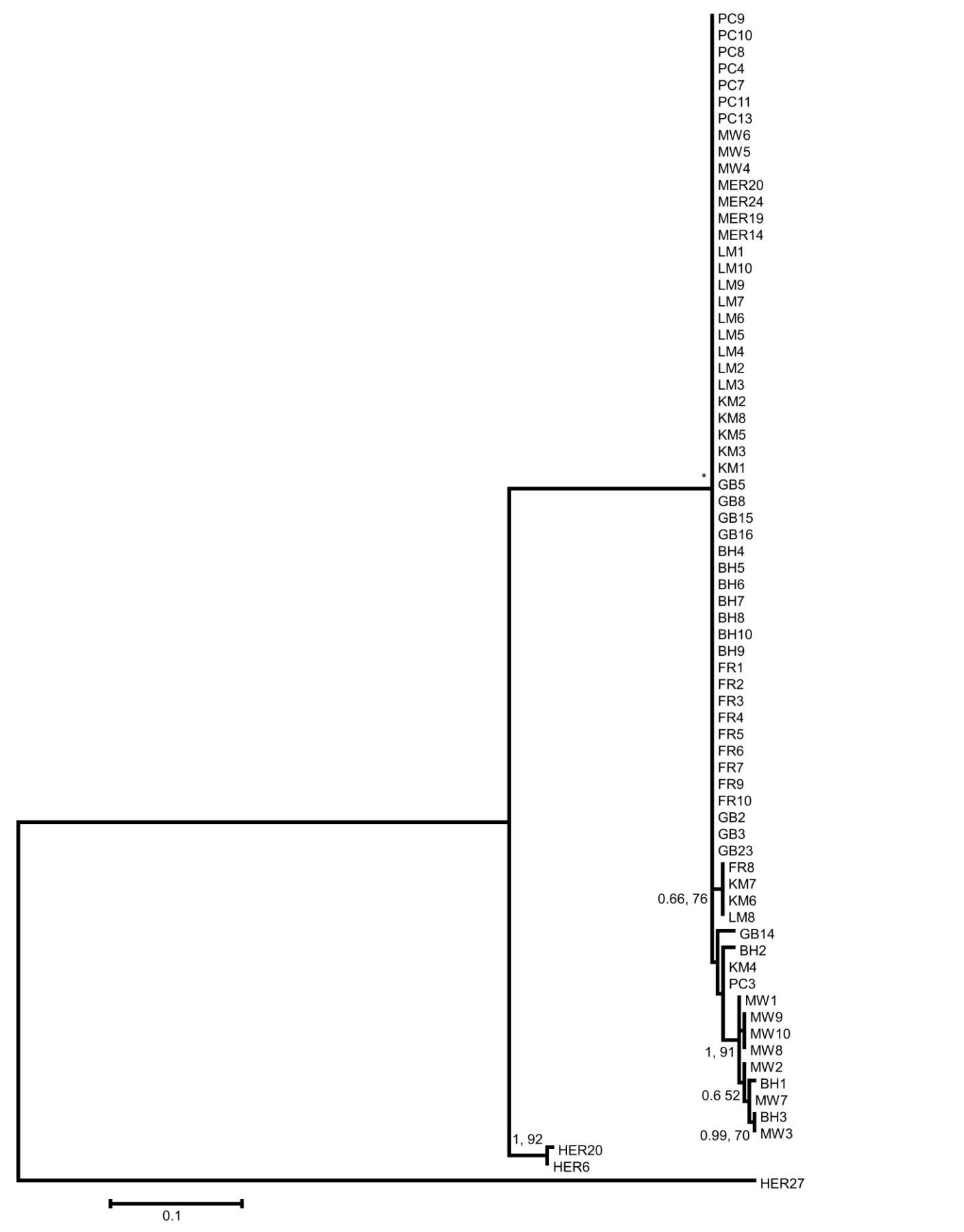


Supplementary Fig. S1B: Maximum Likelihood (ML) phylogenetic tree of various *Ostreopsis* cf. *siamensis* strains based on D1-D3 LSU rDNA region. Numbers at nodes represent posterior probabilities from Bayesian Inferences (BI) and bootstrap support values from ML based on 1000 pseudo-replicates. Only bootstrap values > 50% are shown.

* represents 1, 100 support values for BI and ML respectively.


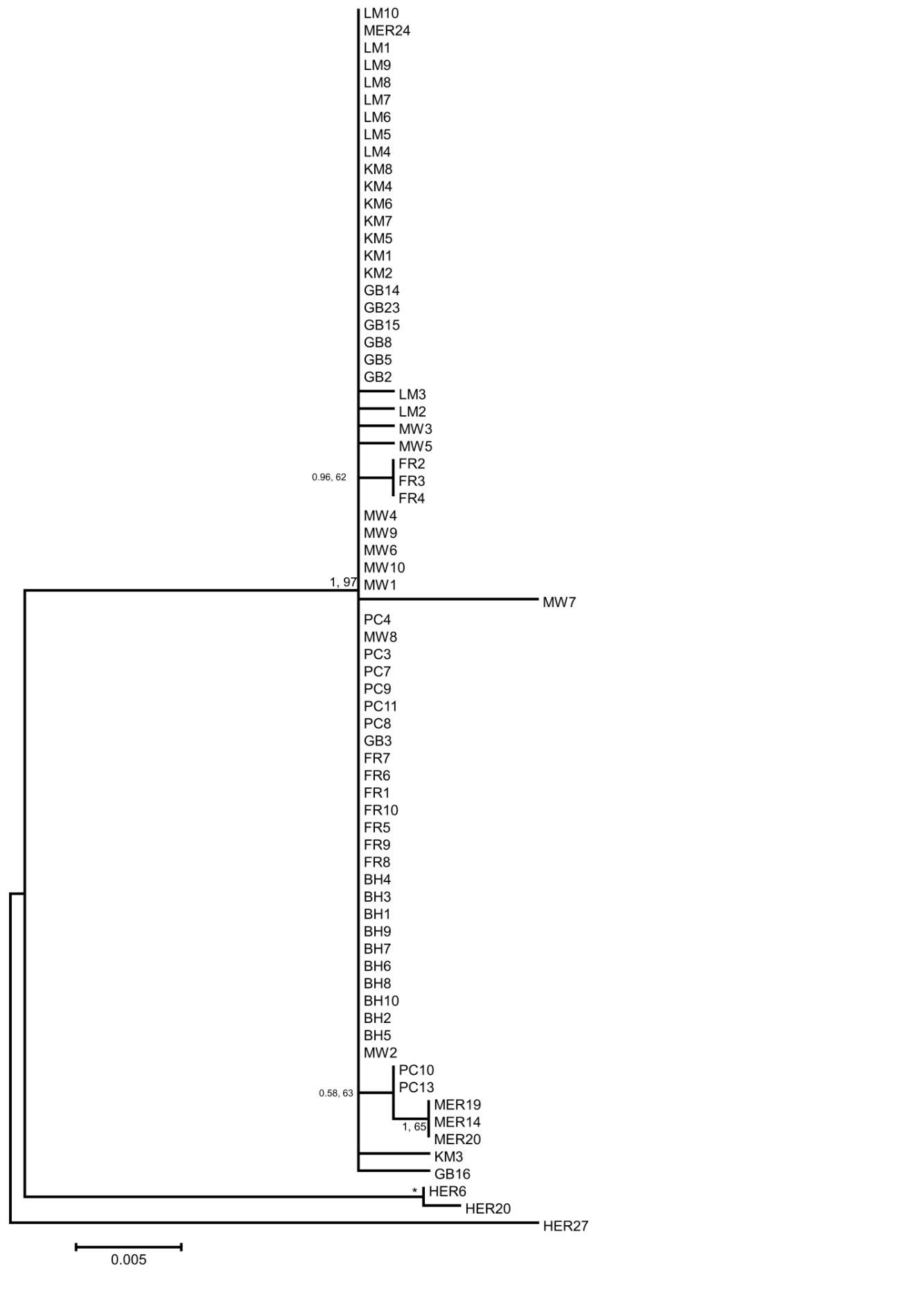


Supplementary Fig. S1C: Maximum Likelihood (ML) phylogenetic tree of various *Ostreopsis* cf. *siamensis* strains based on D8-D10 LSU rDNA region. Numbers at nodes represent posterior probabilities from Bayesian Inferences (BI) and bootstrap support values from ML based on 1000 pseudo-replicates. Only bootstrap values > 50% are shown.

* represents 1, 100 support values for BI and ML respectively.
