# Supplementary material for: Functional significance of phylogeographic structure in a toxic benthic marine microbial eukaryote over a latitudinal gradient along the East Australian Current
Source: Ecol Evol. 2020 May 21;10(13):6257–73. doi: 10.1002/ece3.6358 (PMC7381561; doi:10.1002/ece3.6358)
Supplement: Supplementary file 2 — Figure S2 [file ECE3-10-6257-s002.docx]

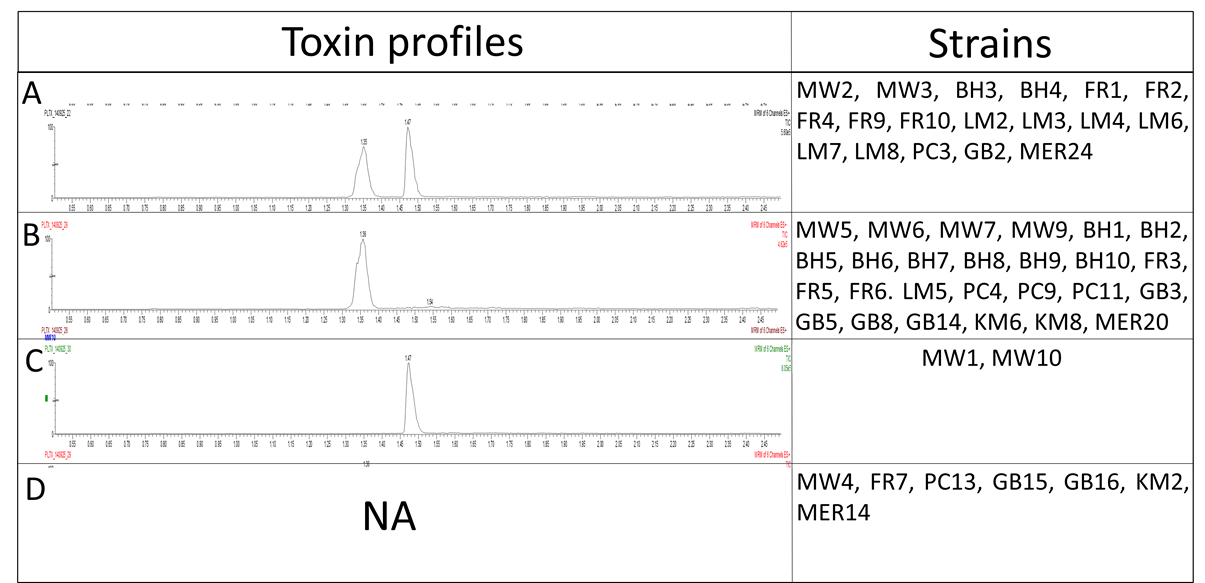


Supplementary Fig. S2: Toxin profile variation amongst *Ostreopsis* cf. *siamensis* strains. **A**: Both amino and amide aldehyde fragments observed; **B**: only amino aldehyde fragment observed; **C**: Only amide aldehyde fragment observed; and **D**: No fragments observed (below the limit of detection).
