# Supplementary material for: Functional significance of phylogeographic structure in a toxic benthic marine microbial eukaryote over a latitudinal gradient along the East Australian Current
Source: Ecol Evol. 2020 May 21;10(13):6257–73. doi: 10.1002/ece3.6358 (PMC7381561; doi:10.1002/ece3.6358)
Supplement: Supplementary file 3 — Figure S3 [file ECE3-10-6257-s003.docx]

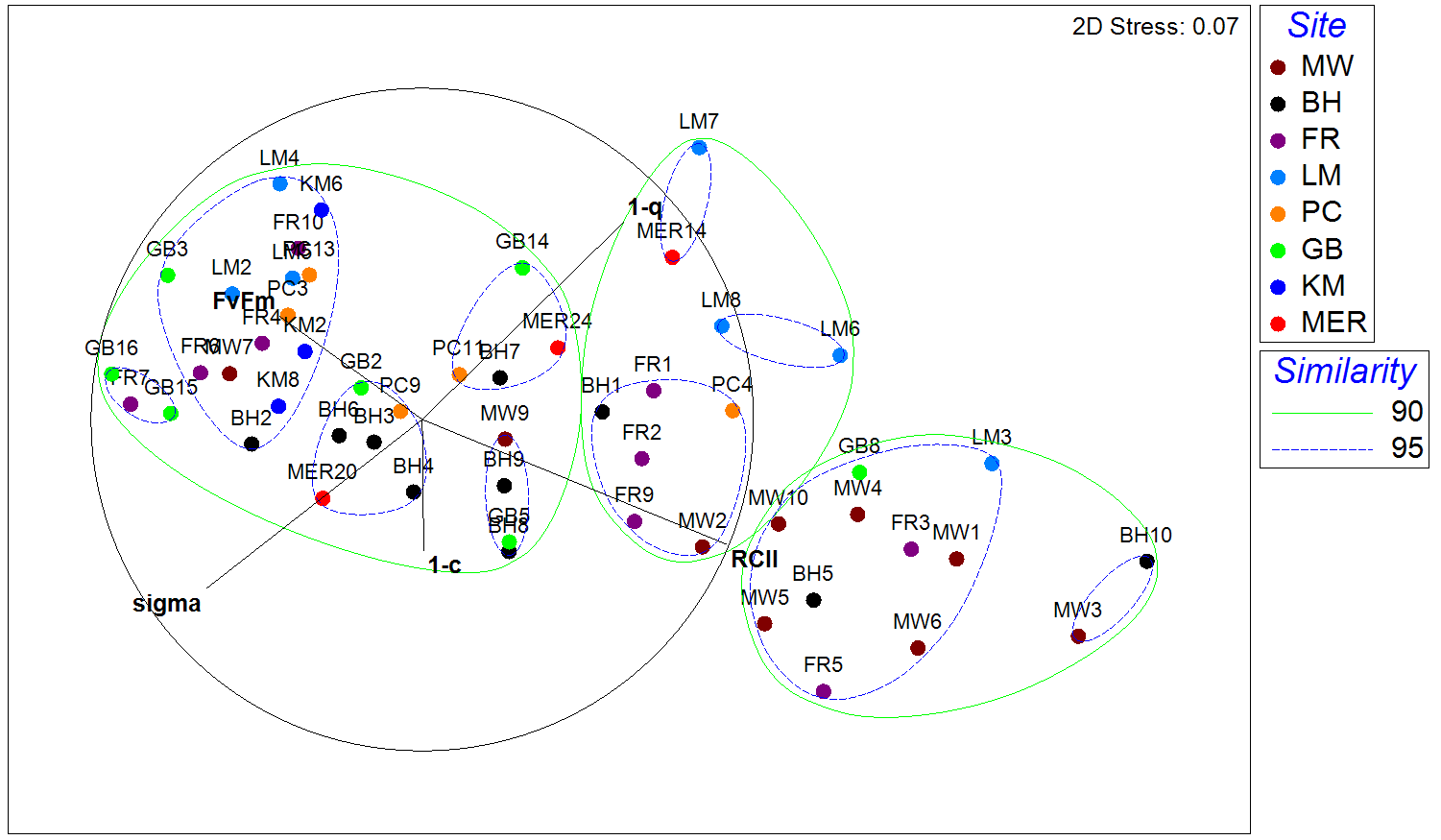


Supplementary Fig. S3: Cluster analysis and multidimensional scaling (MDS) based on factors regulating the ETR, i.e. σ, F*v*/F*m*, cellular [RCII] concentration, (1 – C) and (1 – Q). Similarity is shown at the 90 and 95% levels and vectors driving the clustering are shown in black.
