# Supplementary material for: Functional significance of phylogeographic structure in a toxic benthic marine microbial eukaryote over a latitudinal gradient along the East Australian Current
Source: Ecol Evol. 2020 May 21;10(13):6257–73. doi: 10.1002/ece3.6358 (PMC7381561; doi:10.1002/ece3.6358)
Supplement: Supplementary file 4 — Figure S4 [file ECE3-10-6257-s004.docx]

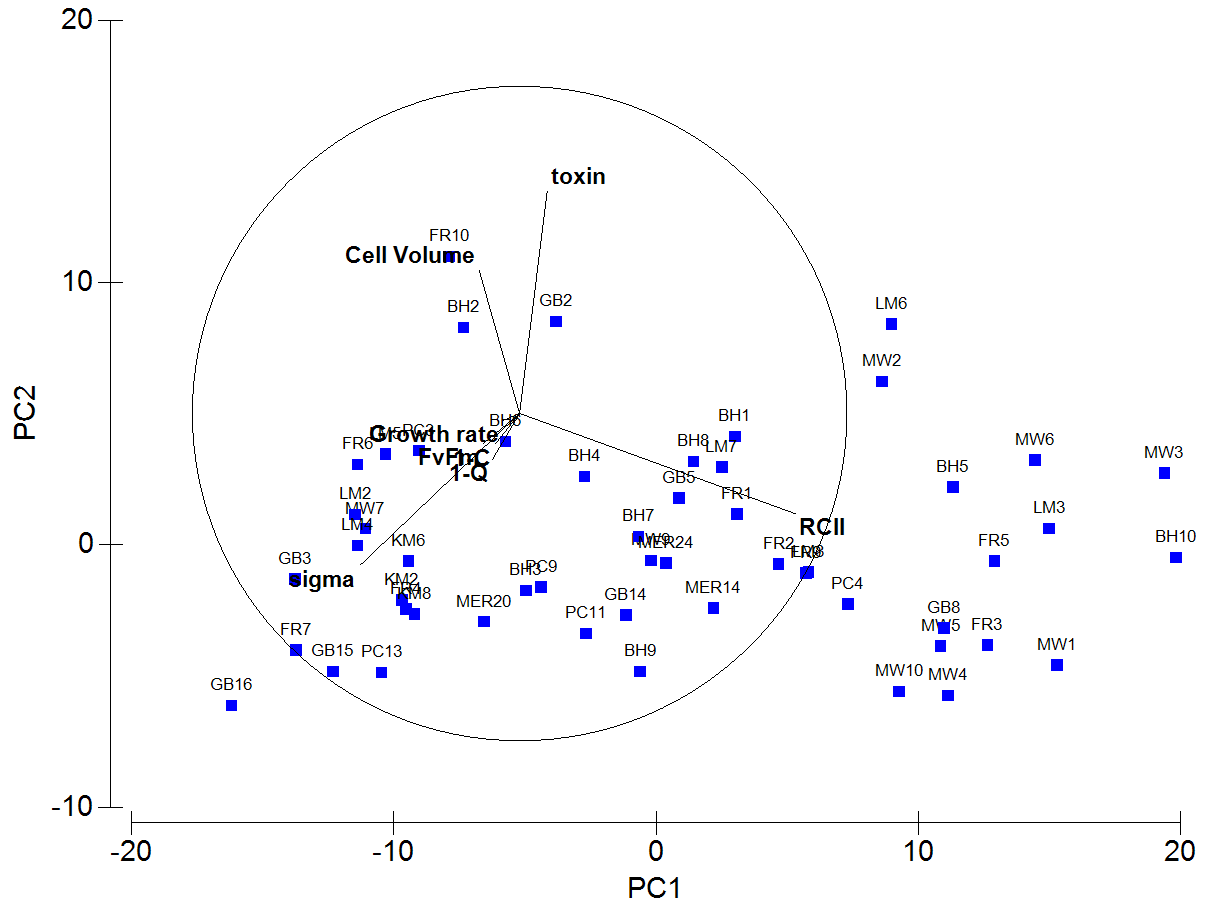


Supplementary Fig. S4: Two-dimensional principal component analysis of phenotypic variables in *Ostreopsis* cf. *siamensis* clones. Light harvesting (F*v*/F*m*, σ, [RCII] concentration and light utilization, i.e. (1-C and (1-Q), along with cell volume, growth rates and amount of PLTX-like compounds were normalised across all strains and indicated in black bars.
