# Supplementary material for: Functional significance of phylogeographic structure in a toxic benthic marine microbial eukaryote over a latitudinal gradient along the East Australian Current
Source: Ecol Evol. 2020 May 21;10(13):6257–73. doi: 10.1002/ece3.6358 (PMC7381561; doi:10.1002/ece3.6358)
Supplement: Supplementary file 5 — Table S1 [file ECE3-10-6257-s005.docx]

Supplementary Table S1: Primers used for PCRs and their annealing temperatures (T_a_).

| **Primer Name** | **Primer sequence** | **Target region** | **Direction** | **T_a_ (^°^C)** | **Reference** |
| --- | --- | --- | --- | --- | --- |
| D1R | 5'-ACC CGC TGA ATT TAA GCA TA-3' | D1-D3 | Forward | 56 | Scholin, Herzog, Sogin, & Anderson, 1994 |
| D3B | 5'-TCG GAG GGA ACC AGC TAC TA-3' | D1-D3 | Reverse | 56 | Nunn, Theisen, Christensen, & Arctander, 1996 |
| FD8/D8 | 5'-GGA TTG GCT CTG AGG GTT GGG-3' | D8-D10 | Forward | 62 | Chinain, Faust, & Pauillac, 1999 |
| RB/D10 | 5'-GAT AGG AAG AGC CGA CAT CGA-3' | D8-D10 | Reverse | 62 | Chinain et al., 1999 |
| GLD8_421F | 5'-ACA GCC AAG GGA ACG GGC TT-3' | D8-D10 | Forward | 62 | Nishimura et al., 2013 |
| GLD8_677R | 5'-TGT GCC GCC CCA GCC AAA CT-3' | D8-D10 | Reverse | 62 | Nishimura et al., 2013 |
| ITS A | 5'-GTA ACA AGG THT CCG TAG GT-3' | **ITS/5.8S** | Forward | 56 | LaJeunesse, 2001 |
| ITS B | 5'-AKA TGC TTA ART TCA GCR GG-3' | **ITS/5.8S** | Reverse | 56 | LaJeunesse, 2001 |

References:

Chinain, M., Faust, M. A., & Pauillac, S. (1999). Morphology and molecular analyses of three toxic species of *Gambierdiscus* (Dinophyceae): *G. pacificus*, sp. nov., *G. australes*, sp. nov., and *G. polynesiensis*, sp. nov. *Journal of Phycology, 35*, 1282-1296.

LaJeunesse, T. C. (2001). Investigating the biodiversity, ecology, and phylogeny of endosymbiotic dinoflagellates in the genus *Symbiodinium* using the ITS region: in search of a “species” level marker. *Journal of Phycology, 37*, 866-880.

Nishimura, T., Sato, S., Tawong, W., Sakanari, H., Uehara, K., Shah, M. M. R., . . . Yamaguchi, H. (2013). Genetic diversity and distribution of the ciguatera-causing dinoflagellate *Gambierdiscus* spp.(Dinophyceae) in coastal areas of Japan. *PloS one, 8*, e60882.

Nunn, G., Theisen, B., Christensen, B., & Arctander, P. (1996). Simplicity-correlated size growth of the nuclear 28S ribosomal RNA D3 expansion segment in the crustacean order Isopoda. *Journal of Molecular Evolution, 42*, 211-223.

Scholin, C. A., Herzog, M., Sogin, M., & Anderson, D. M. (1994). Identification of group- and strain- specific genetic markers for globally distributed *Alexandrium* (dinophyceae). Sequence analysis of a fragmetn of the LSU rRNA gene. *Journal of Phycology, 30*, 999-1011.
